# Long intergenic non-coding LINC00657 regulates tumorigenesis of glioblastoma by acting as a molecular sponge of miR-190a-3p

**DOI:** 10.18632/aging.101845

**Published:** 2019-03-05

**Authors:** Liangzhao Chu, Lei Yu, Jian Liu, Shibin Song, Hua Yang, Feng Han, Fen Liu, Yaxin Hu

**Affiliations:** 1 Department of Neurosurgery, Hospital affiliated to Guizhou Medical University, Guiyang 550004, Guizhou, China; 2 Prenatal Diagnosis Center, Hospital affiliated to Guizhou Medical University, Guiyang 550004, Guizhou, China; * Equal contribution

**Keywords:** LINC00657, glioblastoma, PTEN, miR-190a-3p

## Abstract

To detect the aberrantly expressed long non-coding RNAs in glioblastoma, two pairs of glioblastoma and adjacent normal tissues were firstly analyzed by RNA sequencing. Long intergenic non-coding RNA LINC00657 was considered to play a vital role in glioblastoma based on the results of RNA sequencing. Hence, we aimed to investigate the mechanisms by which LINC00657 regulated the tumorigenesis of glioblastoma. The level of LINC00657 in 40 glioblastoma samples and glioblastoma cell lines was detected by RT-qPCR. LINC00657 was significantly decreased in patients with glioblastoma compared with adjacent normal tissues. Overexpression of LINC00657 inhibited proliferation, colony formation, invasion and migration in glioma cells via inducing apoptosis. Dual luciferase report assay indicated LINC00657 was the target of miR-190a-3p. Overexpression of LINC00657 greatly inhibited the relative amount of miR-190a-3p. Besides, miR-190a-3p was found to be a negative regulator of PTEN. Additionally, active-caspase 3 was increased in cells transfected with pcDNA3.1-LINC00657. Finally, *in vitro* results were further confirmed by *in vivo* studies using nude mice bearing with glioblastoma tumors. In conclusion, LINC00657 was effective in inhibiting glioblastoma by acting as a molecular sponge of miR-190a-3p to regulate PTEN expression. Therefore, targeting LINC00657 may serve as a potential strategy for the treatment of patients with glioblastoma.

## Introduction

Glioma is considered to be the most common brain tumor in adults whose occupancy in primary neoplasms of the central nervous system (CNS) is nearly 70% [1]. Based on the definitions of World Health Organization (WHO), glioblastoma (GBM), which is known as WHO grade IV astrocytoma, accounts for about 55% of the adult diffuse glioma patient population [1]. Diagnostic biopsy or surgical is normally used as the first method for therapy. Then, chemotherapy and adjuvant radiation will be performed in the second step [2]. However, there are so many difficulties in complete resection. Furthermore, GBM is not sensitive to chemo-/radio- therapeutic agents [3]. The median survival of primary GBM is consider to be approximately 15 months [1]. Therefore, there is a great need to investigate the molecular mechanisms involved in GBM.

For human beings, the human genome is totally transcribed. Interestingly, the occupancy of protein-coding genes is about 2%. In addition, the rest of transcripts are known as non-coding RNAs containing long non-coding RNAs (lncRNAs) and microRNAs (miRNAs) [4]. LncRNAs are defined as the RNA molecules without the ability to code protein and with more than 200 nucleotides (nt) [4]. There is an increasing number of the reports which suggest that lncRNAs are related to epigenetic, genetic and post-transcriptional regulation. More and more studies prove lncRNAs are involved in variety of significant biological phenomena including cancer [4–7].

As a tumor suppressor protein, phosphatase and tensin homolog, PTEN in short, was considered to be an important regulator in tumor cell apoptosis [8–11]. There are several studies about the role of PTEN in GBM. It is considered that PTEN inhibit tumor cell growth and promotes cell apoptosis through PI3K/Akt/mTOR pathway [8–11].

LINC00657 is considered to be important in the initiation and progression of different kinds of cancers. Liu et al. has found that knockdown expression of LINC00657 greatly inhibits tumor cell growth and proliferation which suggests LINC00657 might be an oncogene in breast cancer [4]. Otherwise, Hu et al. has reported that LINC00657 is lowly expressed in hepatocellular carcinoma. Overexpression of LINC00657 could inhibit hepatocellular tumor growth [6]. However, the detailed effects of LINC00657 in the initiation and progression of GBM remain greatly unknown.

In this study, lncRNAs LINC00657 was found to be significantly downregulated in GBM tissues base on the RNA sequencing data. In addition, LINC00657 acted as a ceRNA and inhibited the progression of GBM via modulating the expression of the miR-190a-3p. Furthermore, the LINC00657-miR-190a-3p-PTEN axis was investigated as a novel molecular mechanism in GBM, which provide a new direction for the treatment of GBM.

## RESULTS

### DEGs screened in GBM and adjacent tissues

**Figure 1:**
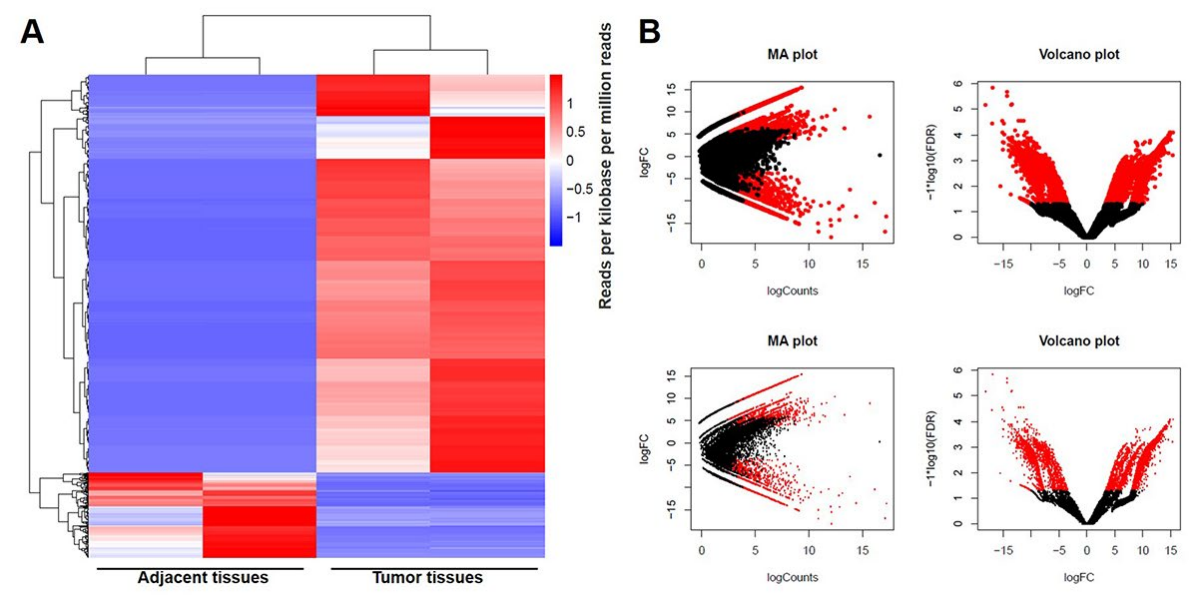
**DEGs screened in GBM and adjacent tissues.** (**A**) The distribution of genes in normal tissues and glioblastoma tissues screened by RNA sequencing using clustering analysis. (**B**) The distribution of genes whose change in expression of false discovery rate (FDR) < 0.01 was marked in red on the MA plot (log total counts versus log2 fold-change). The distribution of genes with a change in expression of log2 fold-change (FC) ≥ 4 was represented in red on the volcano plot (log2 fold-change versus log FDR).

1545 downregulated lncRNAs and 7254 upregulated lncRNAs were observed between GBM and adjacent tissues using RNA sequencing technique. Meanwhile, 2027 downregulated mRNAs and 2120 upregulated mRNAs were picked up. DEGs were analyzed using clustering analysis which were marked in red (Fig. 1A). In addition, DEGs between GBM and adjacent tissues were recorded in MA plot and Volcano plot with the criteria of FDR < 0.01 or log2 fold-change (FC) ≥ 4 which were marked in red (Fig. 1B). Among these DEGs, LINC00657 was one of differentially expressed lncRNAs with >10 (Table 1), and was reported to have a close relationship with tumorigenesis and development [4,6]. Therefore, we focus on investigating the function of LINC00657 in GBM at present study.

**Table 1:** Top differentially expressed lncRNAs with >10 in 2 pairs glioblastoma tumors compared with adjacent tissue.

Gene ID	Gene name	LogFC
ENSG00000214548	MEG3	-15.0617
ENSG00000257151	PWAR6	-15.0469
ENSG00000263934	SNORD3A	-14.3251
ENSG00000228794	LINC01128	-13.7841
ENSG00000242759	LINC00882	-12.3130
ENSG00000255794	RMST	-12.1891
ENSG00000251562	MALAT1	-10.7678
ENSG00000260032	LINC00657	-10.3474

### Signaling pathway analysis of potential DEGs

**Figure 2:**
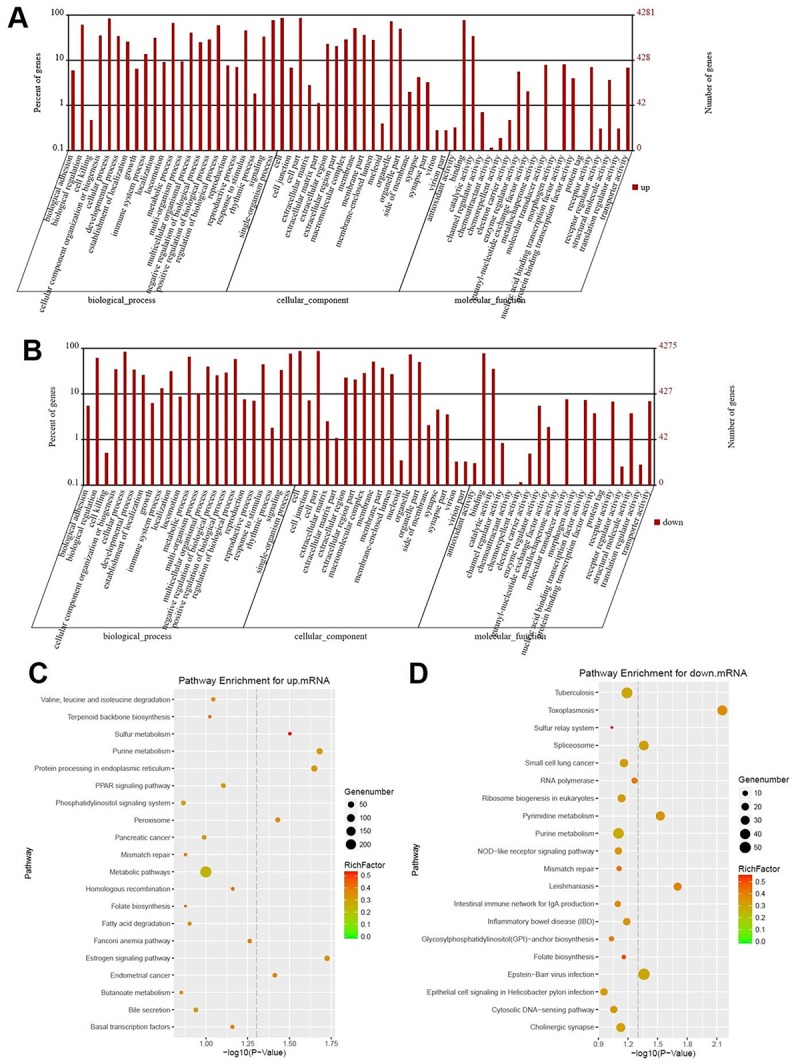
**Signaling pathway analysis of potential DEGs.** (**A**) The biological process, cellular component and molecular function of up-regulated genes were identified by GO analysis. (**B**) The biological process, cellular component and molecular function of down-regulated genes were identified by GO analysis. Items of GO analysis were listed in abscissa axis. The number of genes were recorded in vertical axis. The enriched functions with differential expression genes were picked up according to the criteria that the corrected p-value ≤ 0.05 using Benjamini method. Pathway enrichment for up-regulated mRNA (**C**) and down-regulated mRNA (**D**). Pathway enrichment was executed using KEGG tools. Degree of enrichment was shown in abscissa axis using corrected p values. The color of plot in the figure equaled to rich factor which represented the ratio of genes of the pathway in all the genes of the pathway.

DEGs screened using RNA sequencing were analyzed using GO analysis and KEGG pathway analysis. Results were recorded in Fig. 2. According to GO analysis, biological process of upregulated genes were mainly focused on biological regulation, cellular component organization or biogenesis, cellular process, developmental process, establishment of localization, immune system process, metabolic process, reproductive process and single-organism process (Fig. 2A). Meanwhile, these mentioned biological processes were also observed in downregulated genes (Fig. 2B). Results of KEGG pathway analysis of upregulated mRNA was mainly involved in sulfur metabolism, purine metabolism, protein processing in endoplasmic reticulum, peroxisome, estrogen signaling pathway, endometrial cancer (Fig. 2C). In addition, pathways such as toxoplasmosis, spliceosome, pyrimidine metabolism, leishmaniasis and Epstein-Barr virus infection were obviously detected in downregulated mRNAs using KEGG pathway analysis (Fig. 2D).

### Expression of LINC00657 in GBM tissues and cell lines

**Figure 3:**
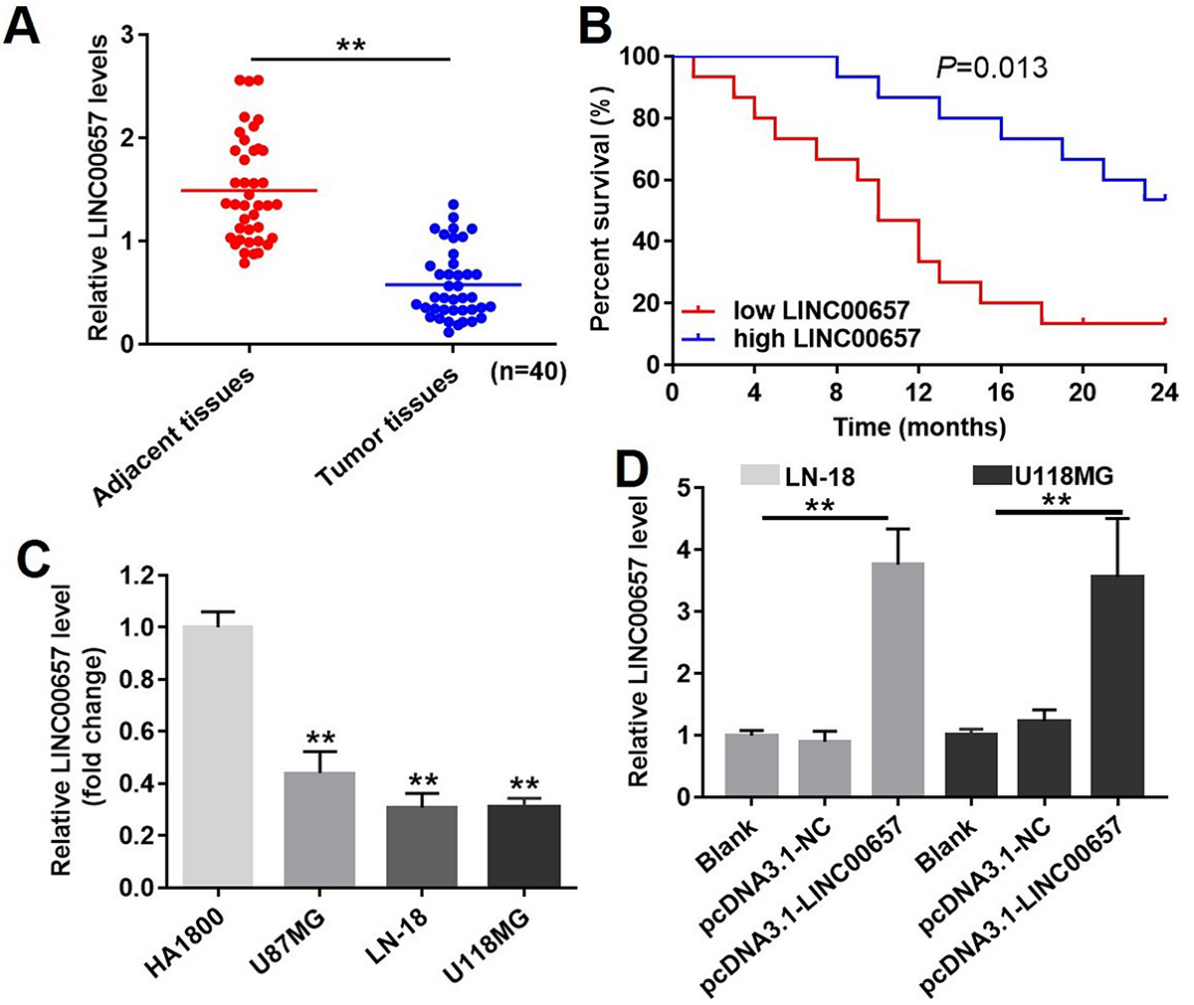
**The expression of LINC00657 in patients with GBM and in GBM cell lines.** (**A**) Relative expression of LINC00657 in normal tissues (adjacent tissues of GBM patients) and tumor tissues of GBM patients (n = 40). **P<0.01, paired t-tests. (**B**) The survival curve of GBM patients with low or high LINC00657. **P=0.013, compared with low LINC00657 group, log-rank test. (**C**) Relative expression of LINC00657 in four kinds of cells including HA1800, U-87MG, LN-18 and U-118MG. **P<0.01, compared with HA1800 group, unpaired t-tests. (**D**) Relative expression of LINC00657 in LN-18 and U-118MG cells after transfecting with pcDNA3.1-NC and pcDNA3.1-LINC00657. Relative expression of LINC00657 was detected using RT-qPCR. GAPHD was chosen to be the internal standard. The mean value of the relative expression of LINC00657 was used as the divide of low and high level of LINC00657. **P<0.01, ANOVA analysis.

The expression of LINC00657 in GBM and adjacent tissues was measured with RT-qPCR. As shown in Fig. 3A, the expression of LINC00657 was significantly decreased in tumor tissues compared with adjacent tissues. Meanwhile, the percent survival in patients with high level of LINC00657 was higher than patients with low LINC00657 (Fig. 3B). In addition, the levels of LINC00657 were negative correlated with tumor size, distant metastasis and WHO stage of patients (Table 2). Next, HA1800 was a kind of normal astrocyte in which the expression of LINC00657 was set as the criteria to calculate the difference in other three GBM cells. As shown in Fig. 3C, the relative expressions of LINC00657 in three GBM cell lines including U-187MG, LN-18 and U-118MG were significantly downregulated compared with HA1800. In order to study the mechanism of LINC00657 in GBM, LN-18 and U-118MG were selected due to the low expression of LINC00657. After transfection with pcDNA3.1-LINC00657, the relative levels of LINC00657 were significantly increased in these two cell lines compared with control group (Fig. 3D). Hence, these two cell lines were proper to be used in the following experiments.

**Table 2:** The relationship between LINC00657 and clinic-pathological parameters of patients with GBM.

**Parameters**	**Number**	**LINC00657**	***p* value**
**Gender**			0.198
Male	22	0.510 ± 0.296	
Female	18	0.648 ± 0.367	
**Tumor volume**			
≤ 3 cm	12	0.875 ± 0.249	0.001**
> 3 cm	28	0.481 ± 0.304	
**Age**			0.414
≤ 50	11	0.536 ± 0.320	
> 50	29	0.592 ± 0.301	
**Distant metastasis**			0.024*
Yes	15	0.488 ± 0.307	
No	25	0.727 ± 0.326	
**WHO stage**			0.002**
I-II	18	0.719 ± 0.325	
III- IV	22	0.473 ± 0.294	

Student’s t test, *P<0.05, **P<0.01.

### Overexpression of LINC00657 inhibited viability and colony formation in GBM cells via enhancing cell apoptosis

**Figure 4:**
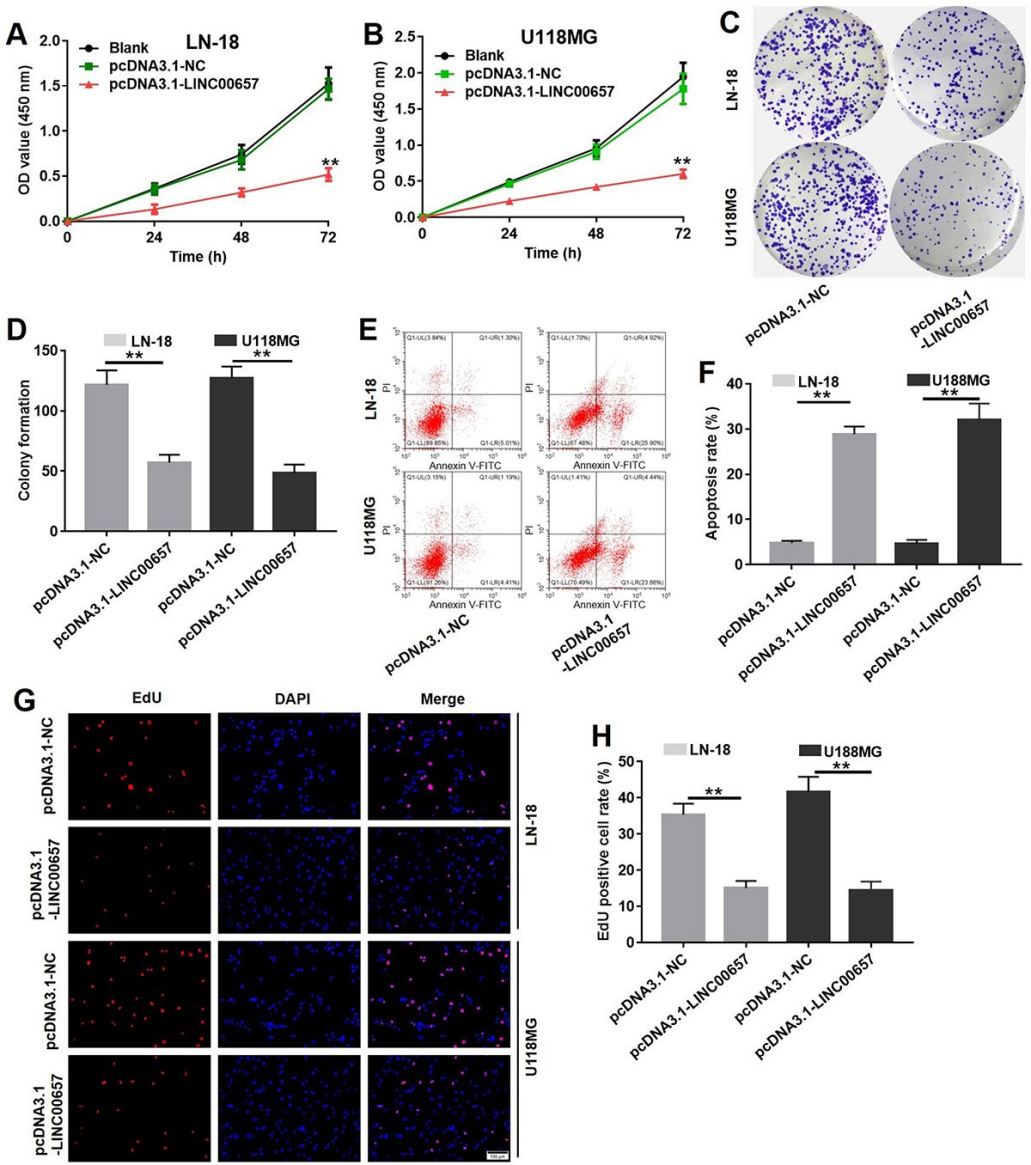
**Overexpression of LINC00657 inhibited viability and colony formation in GBM cells via enhancing cell apoptosis.** Cell viability of LN-18 (**A**) and U-118MG (**B**) after transfecting with pcDNA3.1-NC or pcDNA3.1-LINC00657 for 48 h was detected with CCK-8 assay. Values were represented as mean ± SD. **P<0.01, compared with blank group, ANOVA analysis. (**C**) Cell colony formation stained with crystal violet of LN-18 and U-118MG after transfecting with pcDNA3.1-NC or pcDNA3.1-LINC00657. (**D**) The calculated value of stained cells in cell colony formation of LN-18 and U-118MG after transfecting with pcDNA3.1-NC or pcDNA3.1-LINC00657. (**E**) Cell apoptosis of LN-18 and U-118MG after transfecting with pcDNA3.1-NC or pcDNA3.1-LINC00657 was detected with flow cytometry. (**F**) Apoptosis rate of LN-18 and U-118MG after transfecting with pcDNA3.1-NC or pcDNA3.1-LINC00657. (**G**) LN-18 and U-118MG proliferation after transfecting with pcDNA3.1-NC or pcDNA3.1-LINC00657 using EdU and DAPI staining. (**H**) EdU positive cell rate of LN-18 and U-118MG after transfecting with pcDNA3.1-NC or pcDNA3.1-LINC00657. At least 3 randomly observed fields were chosen to calculate the rate in each group. **P<0.01, compared with control group, unpaired t-tests.

Compared with control group, cell viabilities of LN-18 and U-118MG were remarkably inhibited by LINC00657 overexpression (Fig. 4A and B). Besides, overexpression of LINC00657 also significantly inhibited LN-18 and U-118MG cell colony formation (Fig. 4C and D). In contrast, cell apoptosis rate of LN-18 and U-118MG were greatly increased after pcDNA3.1-LINC00657 transfection (Fig. 4E and F). In addition, the result of EdU staining confirmed cell proliferation in pcDNA3.1-LINC00657 group was highly decreased compared with control group (Fig. 4G and H).

### Overexpression of LINC00657 inhibited cell migration and invasion

**Figure 5:**
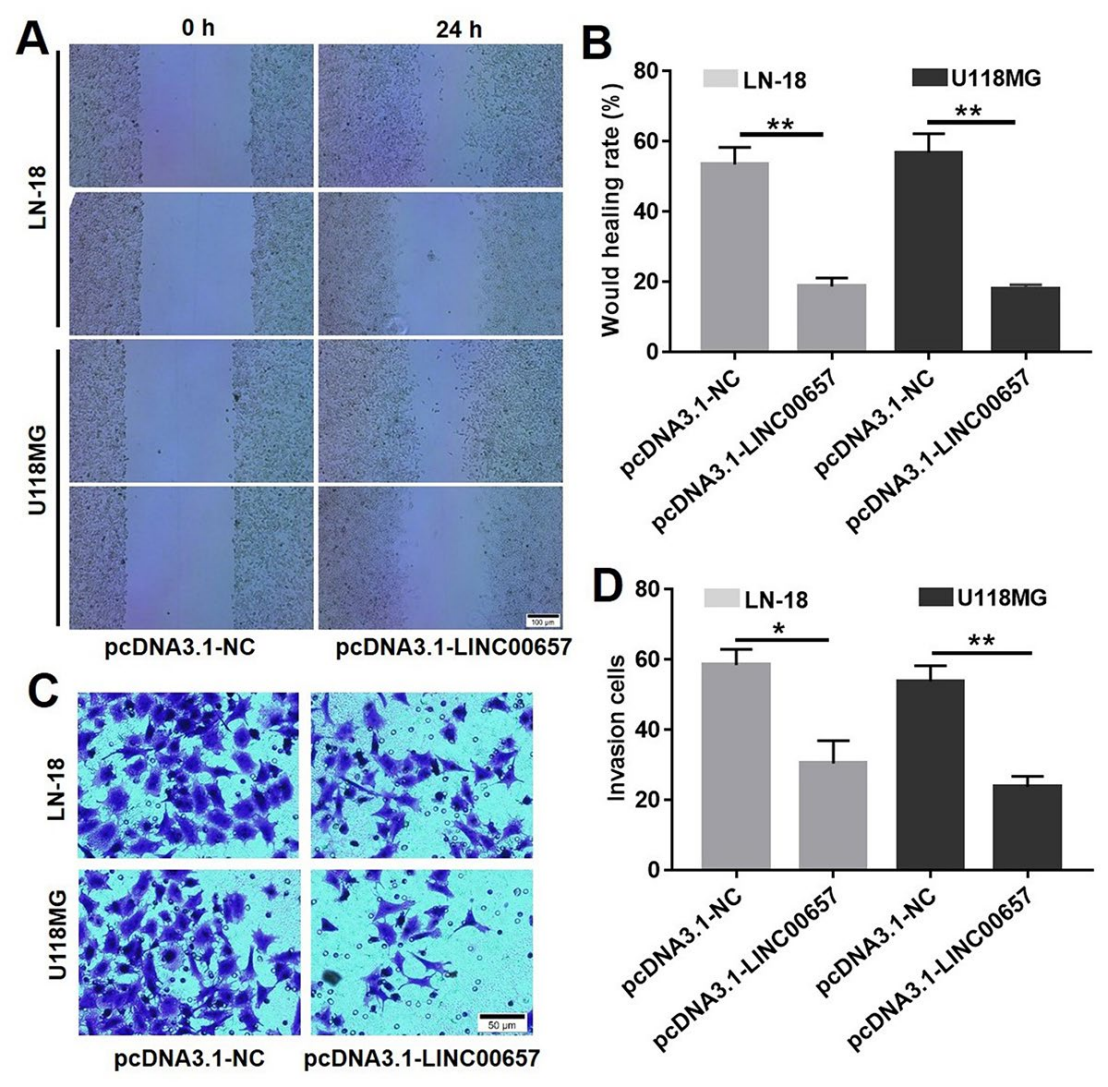
**Overexpression of LINC00657 inhibited cell migration and invasion.** Wound healing assay (**A**) and wound healing rate (**B**) of LN-18 and U-118MG after transfecting with pcDNA3.1-NC or pcDNA3.1-LINC00657. (**C, D**) Cell invasion of LN-18 and U-118MG after transfecting with pcDNA3.1-NC or pcDNA3.1-LINC00657. *P<0.05, **P<0.01, compared with control group, unpaired t-tests.

Wound healing assay was used to evaluate the effect of LINC00657 on cell migration. As shown in Fig. 5A and B, wound healing rates of LN-18 and U-118MG transfected with pcDNA3.1-LINC00657 were obviously decreased compared with control group, which indicated LINC00657 inhibited cell migration. Meanwhile, crystal violet positive staining cells were significantly decreased after treated with pcDNA3.1-LINC00657 in both LN-18 and U-118MG (Fig. 5C). In order to scientifically calculate the invasion cells, 3 different views of each sample in every group were numbered under a light microscope. As shown in Fig. 5D, invasion cells in both LN-18 and U-118MG were remarkably decreased after transfected with pcDNA3.1-LINC00657 compared with control group.

### LINC00657 was a target of miR-190a-3p

**Figure 6:**
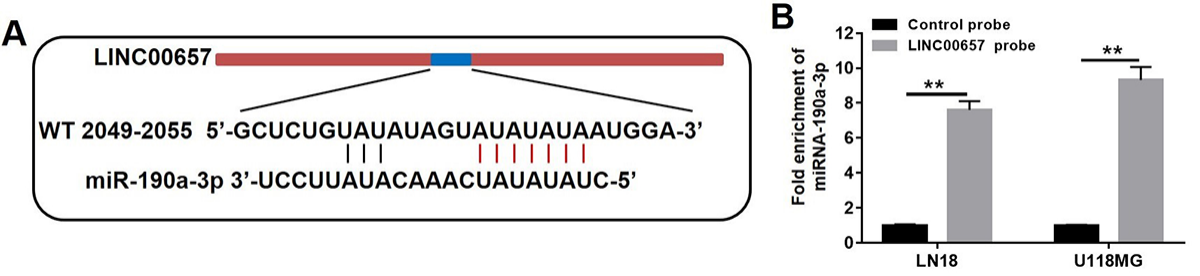
**LINC00657 was a target of miR-190a-3p.** (**A**) The predicted combined sites of LINC00657 and miR-190a-3p. (**B**) Fold enrichment of miR-190a-3p after adding LINC00657 probe in pull-down assay. **P<0.01, compared with control probe group, unpaired t-tests.

By means of bioinformatics analysis (miRanda), there were five miRNA binding sites which were represented in LINC00657 cDNA. The five potential miRNAs were miR-6740-3p, miR-4789-5p, miR-190a-3p, miR-608 and miR-202-5p. It was revealed that binding sites of miR-190a-3p was located in LINC00657 according to the recognition sequences (Fig. 6A). Furthermore, pull down assay was executed to confirm whether LINC00657 was the target of miR-190a-3p. As shown in Fig. 6B, miR-190a-3p was precipitated by LINC00657 probe. All in all, these results confirmed that miR-190a-3p could directly bind to LINC00657 at the miRNA recognition site.

### LINC00657 enhanced cell apoptosis via indirectly regulating PTEN pathway

**Figure 7:**
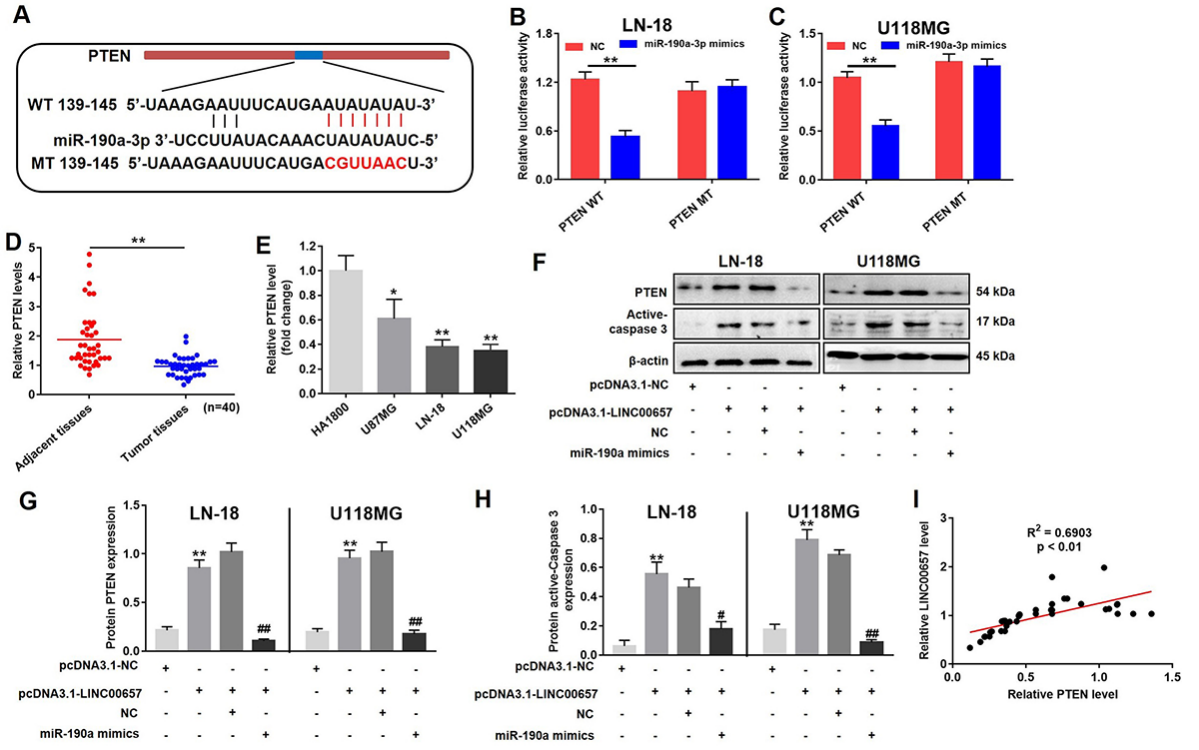
**LINC00657 enhanced cell apoptosis through indirectly regulating PTEN.** (**A**) The predicted combined sites of PTEN and miR-190a-3p. Relative luciferase activity in LN-18 (**B**) and U-118MG (**C**) transfected with PTEN WT or PTEN MT following treatment with NC or miR-190a-3p mimics. **P<0.01, compared with NC group, unpaired t-tests. (**D**) Relative PTEN levels in normal tissues (adjacent tissues of GBM patients) and GBM tumor tissues (n = 40) were detected using RT-qPCR. **P<0.01, compared with adjacent tissues, paired t-tests. (**E**) Relative PTEN levels in in four kinds of cells including HA1800, U-87MG, LN-18 and U-118MG. *P<0.05, **P<0.01, compared with HA1800 group, unpaired t-tests. (**F**) Relative expressions of PTEN and active-caspase 3 in LN-18 and U-118MG transfected with pcDNA3.1-LINC00657 or pcDNA3.1-NC following treatment with NC or miR-190a-3p mimics. (**G, H**) Relative expressions of PTEN and active-caspase 3 in LN-18 and U-118MG cells were quantified by Image-Pro Plus. (**I**) Pearson's correlation scatter plot of the expressions of LINC00657 and PTEN in NSCLC tumor tissues. **P<0.01, compared with control group. ^#^P<0.05, ^##^P<0.01, compared with pcDNA3.1-LINC00657, unpaired t-tests.

With the aid of bioinformatics analysis, PTEN was considered to be the target of miR-190a-3p (Fig. 7A). In order to further confirm the relationship between PTEN and miR-190a-3p, the dual-luciferase reporter assay was performed. Overexpression of miR-190a-3p significantly reduced the relative luciferase activity in PTEN WT group while did not affect the relative luciferase activity in PTEN MT group (Fig. 7B). Furthermore, the relative PTEN level in GBM tissues was notably downregulated compared with adjacent tissues (Fig. 7D). Similar to previous results, the relative PTEN level in three GBM cells including U-187MG, LN-18 and U-118MG were much lower than that in HA1800 (Fig. 7E). The results of western blot indicated the levels of PTEN and active-caspase3 were significantly increased in cells treated with pcDNA3.1-LINC00657 compared with control group, which were notably reversed by miR-190a-3p mimics (Fig. 7F, G and H). In addition, the level of LINC00657 was positive correlation with the expression of PTEN (Fig. 7I).

**Figure 8:**
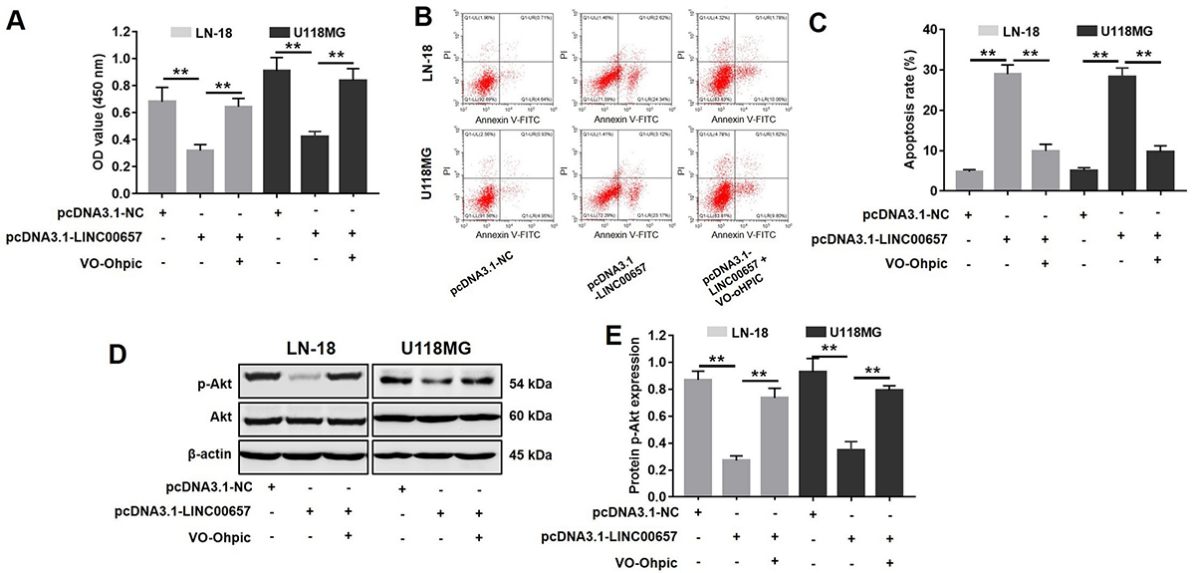
**Inhibitory effects of LINC00657 on GBM tumor growth was reversed by VO-Ohpic.** (**A**) Cell viability of LN-18 and U-118MG after transfecting with pcDNA3.1-LINC00657 with or without VO-Ohpic (100 nM) for 48 h was detected CCK-8 assay. (**B**) Cell apoptosis of LN-18 and U-118MG was detected with flow cytometry. (**C**) Apoptosis rate of LN-18 and U-118MG. (**D**) The protein level of p-Akt in GBM cells after transfecting with pcDNA3.1-LINC00657 with or without VO-Ohpic for 48 h was measured with western blot. (**E**) Relative p-Akt levels in the cells. **P<0.01.

PTEN selective inhibitor VO-Ohpic was used to further confirm the role of PTEN during GBM tumorigenesis. As indicated in Fig. 8A-C, pcDNA3.1-LINC00657 induced GBM cell growth inhibition and apoptosis were significantly reversed by VO-Ohpic. Additionally, the results of western blot indicated the levels of p-Akt were significantly increased in cells treated with pcDNA3.1-LINC00657 compared with control group, which were dramatically reversed by VO-Ohpic as well. Taken together, LINC00657 inhibited the expression of miR-190a-3p via indirectly increasing the level of PTEN.

### The effects of LINC00657 on GBM tumor growth

**Figure 9:**
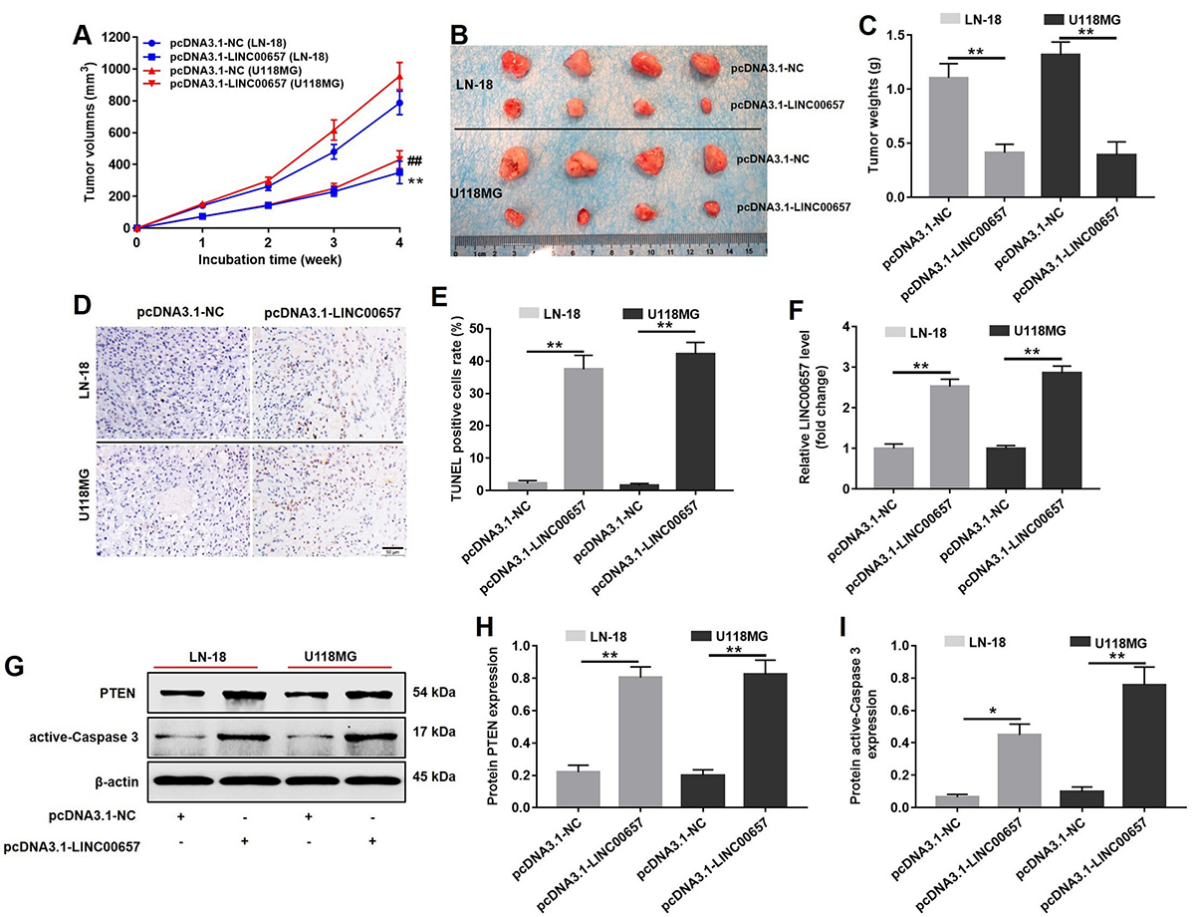
***In vivo* study on inducing cell apoptosis effect of LINC00657.** (**A**) The pcDNA3.1-LINC00657 transfected LN-18 and U-118MG cells were subcutaneously injected to nude mice, and the tumor volume were measured weekly. The tumor volume was equal to length × width^2^ × 0.5. (**B**) Tumors were isolated from xenografts in 4 weeks. (**C**) Tumor weights in each group were quantified. (**D**) TUNEL staining of tumor tissues in each group. (**E**) TUNEL positive cell rate in each group was quantified. (**F**) Relative level of LINC00657 in each group was detected with qPCR. (**G**) Relative expressions of PTEN and active-caspase 3 in tumors were detected with western blot assay. (**H, I**) Relative expressions of PTEN and active-caspase 3 in tumors were quantified with Image-Pro Plus. *P<0.05, **P<0.01.

Animal bearing with transplanted tumors was performed to study the effect of LINC00657 on GBM tumor growth *in vivo*. As shown in Fig. 9A, B and C, the tumor volumes and tumor weights of animals were significantly decreased in pcDNA3.1-LINC00657 group, compared with control group. According to Fig. 9D and E, TUNEL positive cells rate in group transfected with pcDNA3.1-LINC00657 were much higher than that in control group. In addition, the result of PCR indicated the relative level of LINC00657 was notably upregulated in pcDNA3.1-LINC00657 group, compared with control group. Furthermore, the relative protein level of PTEN and active-caspase3 was remarkably increased in group transfected with pcDNA3.1-LINC00657 in both animal groups bearing with LN-18 and U-118MG separately (Fig. 9G, H and I). Thus, overexpression of LINC00657 could inhibit GBM tumor growth *in vivo*.

## DISCUSSION

In this study, the LINC00657-miR-190a-PTEN axis was firstly reported in GBM. Overexpression of LINC00657 inhibited the expression of miR-190a-3p. In turn, reduced miR-190a-3p promoted the expression of PTEN which finally regulated caspase3 through PI3K/Akt/mTOR pathway *In vitro* and *in vivo*.

LINC00657 is a novel non-coding RNA which was firstly identified in breast cancer. Compared to normal tissues, LINC00657 is abnormally expressed in breast cancer tissues [4]. However, the clinical value of LINC00657 in GBM is unknown. Here, we systematically studied the potential role of LINC00657 in GBM. Our results demonstrated that the expression of LINC00657 in glioblastoma tissues were notably downregulated compared with adjacent normal tissues. To our knowledge, this is the first study of LINC00657 affecting GBM. Results demonstrated that LINC00657 expression was significantly correlated with cell proliferation, apoptosis, cell invasion and tumor growth. On the other hand, we further clarified that overexpression of LINC00657 inhibited GBM cell migration and invasion. This is the first report to show the functional significance of LINC00657 expression in human GBM. Our results suggested that LINC00657 suppressed the malignant progression of GBM.

Extensive evidence suggests that lncRNAs are essential in the regulatory network of competing endogenous RNAs (ceRNAs), which act as endogenous miRNA sponges by binding to miRNAs and thereby affect their function [14,15]. There is mutual inhibition in the ceRNA network between lncRNA and miRNA [16–18]. To assess whether LINC00657 acts as a miR-190a-3p sponge, we performed the dual luciferase reporter assay to determine whether LINC00657 is a direct target of miR-190a-3p. The results showed that miR-190a-3p significantly inhibited luciferase activity. Thus, LINC00657 was a direct target of miR-190a-3p.

The key role of miRNAs is to modulate the expression of their target genes via mRNA cleavage and/or by inhibiting translation, depending on the degree of complementarity of the 3' UTR of the target genes [19–21]. Furthermore, computational algorithms were used to predict miRNA targets, which are mainly based on the base pairing between 3' UTR of target gene and miRNA. PTEN was predicted as a direct target of miR-190a-3p at its 3'-UTR mRNA by bioinformatics, which was confirmed by a luciferase reporter assay. In the present study LINC00657 acted as a sponge of miR-190a-3p and promoted the expression of PTEN in GBM.

In conclusion, the pleiotropic effect of LINC00657 on GBM tumorigenesis suggested that targeting LINC00657 may serve as a potential strategy for the treatment of patients with GBM.

## MATERIALS AND METHODS

### Clinical samples

40 pairs of GBM tissues and adjacent normal tissues were obtained from patients with GBM in The Affiliated Hospital of Guizhou Medical University from Jan, 3 2014 to Aug, 8 2017. These samples were collected during surgical therapy. Written informed consent was obtained in all cases. The relative expression of LINC00657 and PTEN in GBM tissues were analyzed by RT-qPCR. All experimental protocols were approved by the Affiliated Hospital of Guizhou Medical University Ethics Committee.

### RNA sequencing and data analysis

Two GBM tissues (GBM group) and the two relative adjacent tissues (normal control) were randomly selected in this experiment. The total RNA were extracted by using TRIzol reagent according to the manufacturer’s instruction (Thermo Fisher Scientific, Waltham, MA, USA). The total genomic DNA was removed from RNA sample using DNase I (New England Biolabs, Beijing, China). Then, the RNA sequencing was performed according to the methods reported before [12]. TruSeq RNA library preparation kit (IIIumina, San Diego, CA, USA) based on rRNA depletion was used in this study. Paired end (PE) sequencing model was used and the cycle number was 150. In addition, 2 G reads were produced per sample.

The edge-R package (http://www.bioconductor.org/packages/release/bioc/html/edgeR.html) was used to analyzed Differential expressed genes (DEGs) in tumor and adjacent tissues. Gene ontology (GO) is chosen and used in this study to find the potential gene annotation. In order to analyze the DEGs at the functional level, hypergeometric test was executed to find the biological functions with enriched DEGs (http://mathworld.wolfram.com). P-values obtained from hypergeometric test were corrected by Benjamini. In order to obtain biological processes (BPs), cellular components (CCs) and molecular functions (MFs), online tool DAVID (https://david.ncifcrf.gov/) was used. The criteria used in Go analysis was p-value ≤ 0.05. Kyoto Encyclopedia of Genes and Genomes (KEGG) pathway enrichment analyses were performed using the online tool KOBAS with the criteria of screening p-value ≤ 0.05.

### Cell culture and RT-qPCR

Human astrocyte HA1800 was obtained from the Sciencell (Carlsbad, CA, USA) and cultured in Dulbecco’s modified Eagle’s medium (DEME, Thermo Fisher Scientific, Waltham, MA, USA) with 10% fetal bovine serum (FBS, Thermo Fisher Scientific, Waltham, MA, USA). Human GBM cell lines U-87 MG was purchased from ATCC and cultured in Eagle’s minimum essential medium (EME, Thermo Fisher Scientific, Waltham, MA, USA) with 10% fetal bovine serum (FBS, Thermo Fisher Scientific, Waltham, MA, USA). Human GBM cell lines LN-18 and U-118 MG were both purchased from ATCC and cultured in DMEM added with 10% FBS. PTEN selective inhibitor VO-Ohpic trihydrate (VO-Ohpic) was purchased from MedChem Express (Monmouth Junction, NJ, USA). Other commercially available products were all provided by Dalian Meilun Biotechnology Co., Ltd (Dalian, China).

The total RNA from GBM tissues, NC tissues or cells were extracted by using TRIzol reagent (Thermo Fisher Scientific, Waltham, MA, USA). For lncRNA, reverse transcription was performed with PrimeScript RT reagent Kit with gDNA Eraser (TaKaRa, Japan). For miRNA, reverse transcription was performed using Mir-X™ miRNA First Strand Synthesis Kit (TaKaRa, Japan). Next, real-time PCR was performed as follows: firstly, 94°C for 4 min, secondly, 94°C for 30 s, 50°C for 30 s, finally, 72°C for 40 s. Totally, there were 40 thermal cycles by using SYBR premix Ex Taq II kit (TaKaRa, Dalian, China) on an ABI 7900HT instrument (ABI, New York, NY, USA). The primers for LINC00657, miR-6740-3p, miR-4789-5p, miR-190a-3p, miR-608, miR-202-5p and PTEN were all purchased from Nanjing Decode Genomics BioTech Co., Ltd (Nanjing, China). For clinical sample, the value of the adjacent normal tissue (first patient) was set as 1 and the relative expression of other adjacent tissues and GBM tissues were normalized to this tissue. All samples were performed in triplicates. 2-^ΔΔCT^ method was used to calculate the relative gene expression. GAPHD and U6 were used as internal control for detection of LINC00657 or miR-190a-3p, respectively. LINC00657: forward, 5’-TGATAGGATACATCTTG GACATGGA-3’; reverse 5’ -AACCTAATGAACAAG TCCTGACATACA-3’. MiR-190a-3p: forward, 5’- ACACTCCAACAAACTATATATCGGGTCTC-3’; reverse 5’ -TGGTGATCTGCAGTC-3’. GAPDH: forward, 5’-GTCTCCTCTGACTTCAACAGCG-3’; reverse 5’-ACCACCCTGTTGCTGTAGCCAA-3’. U6: forward, 5’-CTCGCTTCGGCAGCACATATACT-3’; reverse 5’-ACGCTTCACGAATTTGCGTGTC-3’.

### Plasmid construction and cell transfection

The cDNA sequence of LINC00657 was introduced into the pcDNA3.1 expression vector (Invitrogen, shanghai, China) to construct the plasmid complementary DNA LINC00657 cDNA. The miRNAs involved in this study were all purchased from GenePharma (Shanghai, China). LN-18 and U-118 MG cells (2 × 10^5^) were transfected with 5 μg pcDNA3.1-LINC00657 based on the manufacturer’s recommended instructions by using Lipofectamine 2000 Reagent (Thermo Fisher Scientific, Waltham, MA, USA). Cells transfected with pcDNA3.1 were used as the negative controls (NC). Then, stable expression of LINC00657 cell lines (LN-18 and U-118MG) were established by treating with 600 μg/ml neomycin for 4 weeks.

In addition, cells LN-18 and U-118 MG cells (2 × 10^5^) were transfected with 25 nM miR-190a-3p mimics by using Lipofectamine 2000 Reagent. Cells transfected with scramble were used as the NC.

### CCK-8 assay and cell proliferation

The cell viability value was measured by cell counting kit-8 assay (Meilunbio, Dalian, China). Cells (LN-18 and U-118 MG, 2000 cells per well) were cultured in 96-well plates and transfected with pcDNA3.1-NC and pcDNA3.1-LINC00657. The cell viability of cells collected from indicated time points after transfection was determined by using CCK-8 in triplicate. 10 μL CCK-8 reagent was added into the cells and incubated with the cells for another 1 h at 37°C. Next, the resulting product was measured the absorbance at 450 nm using a microplate reader.

The cell proliferation was detected by EdU and DAPI staining. Materials in this assay were all supported by Beyotime Biotechnology (Shanghai, China). LN-18 and U-118 MG cells (1 × 10^4^) were planted into 96-well plates and transfected with pcDNA3.1-NC and pcDNA3.1-LINC00657. Each well was added with 100 μL 50 μM EdU solution and cultured for 2 h. Then, the cells were washed by PBS for twice, 5 min every time. Next, cells were fixed with 4% paraformaldehyde. Apollo® staining reaction solution was added into each well and incubated in a dark circumstance for 30 min. An inverted fluorescence microscope was used to capture the images. For DAPI staining, cells were fixed with 70% ethanol and stained with DAPI (2 μg/mL) for 15 min. Each image shown was representative of 3 randomly observed fields.

### Cell colony formation assay

LN-18 and U-118 MG cells (200 cells per well) were cultured in 6-well plates for about 2 weeks at 37°C in a 5% CO_2_ incubator and transfected with pcDNA3.1-NC and pcDNA3.1-LINC00657. All samples were executed in triplicate. After incubation, the cells were stained with 0.1% crystal violet (Meilunbio, Dalian, China). The number of visible colonies was counted and recorded to calculate the colony formation.

### Cell apoptosis

LN-18 and U-118 MG were transfected with pcDNA3.1-NC or pcDNA3.1-LINC00657 transfected cells for 48 h. Then, the cells were collected and centrifuged at 1000 rpm/min for 5 min. The residue was resuspended with 100 μL binding buffer. 4 μL Annexin V-FITC and 3 μL propidium (PI) were added in the situation. After incubating 15 min at room temperature, 200 μL binding buffer was added and measured by FCM flow cytometry (BD, Bioscience, San Jose, CA, USA).

### Wound healing assay and cell transwell invasion assay

In brief, LN-18 and U-118 MG cells were cultured in 6-well plates and transfected by pcDNA3.1-LINC00657 or NC. After 24 h transfection, the ratio of transfection was nearly 90%. The vertically lineation was scratched on the cell culture plate by using 200 μL pipette tip. After washing with PBS for 3 times, cells were continuing to be cultured in the medium without serum for 24 h. The width of the scarification at different time points (0 h and 24 h) was recorded under light microscope.

The LN-18 and U-118 MG cells invasion was detected by using BioCoat Matrigel Invasion Chambers (Corning New York, NY, USA). The upper chamber is pre-coated with 100 μl of Matrigel for 2 h. After transfection with pcDNA3.1-NC and pcDNA3.1-LINC00657, the LN-18 and U-118 MG cells were added in double to the upper well in serum free medium. Then, the cells were incubated at 37°C for another 24 h. Next, the cells on the surface of the upper chamber membrane were discarded by using cotton swabs. The cells underside of the membrane were fixed in 100% methanol and stained with a solution containing 50% isopropanol, 1% formic acid and 0.5% crystal violet. Then, it was counted under a light microscope (views were randomly selected, at least 5 views per well).

### The dual luciferase reporter system assay

The specific targets between LINC00657 and miR-190a-3p and miR-190a-3p and PTEN were searched separately using three internet-based bioinformatics online softwares, namely TargetScan 6.0, miRanda and miRbase. The target gene sequence and the mutation gene sequence were synthesized by Sangon Biotech (Shanghai). The gene sequence was connected with the carrier psiCHECK-2. After that, the gene sequences was digested by *Xho* I/*Not* I enzyme in order to authenticate the sequence by electrophoresis and sequencing. The psiCHECK2-LINC00657-WT, psiCHECK2-LINC00 657-MT, psiCHECK2-PTEN-WT and psiCHECK2- PTEN-MT were used and connected with hsa-miR-190a-3p mimics separately. Lipo2000 was used in the transfection procedure according to the instruction of Thermo Fisher Scientific. LN-18 or U118MG cells were transfected mimics for 48 h. After transfection, the cells were digested by TritonX-100. Then, the lysate was added with firefly luciferase and renilla luciferase buffer solution and their relative substrate separately. The firefly luciferase was detected according to instruction of manufacture (Promega, Madison, WI, USA).

### Pull-down assay with biotinylated DNA probe

According to the manufacturer’s instruction (Thermo Fisher Scientific, Waltham, MA, USA), the biotinylated DNA probe complementary to LINC00657 was incubated with Dynabeads M-280 Streptavidin after being dissolved in binding and washing buffer for 10 min at room temperature [13]. After incubation, the probe-coated beads were generated. After that, the probe-coated beads were incubated with LN-18 and U-118 MG cell lysates. After incubation, the RNA complexes which were bound to these beads were eluted and extracted for RT-qPCR analysis. The LINC00657 pull-down probe sequence was 5’-AAGACTTACATGGTGCTGTAGA-3’. The negative control was random pull-down probe sequence with the sequence of 5’-AAGACTTTACGAGACGGACTCAA-3’.

### Western Blot

The tumor tissues or cells was lysed with RIPA buffer containing protease inhibitor cocktail (Dalian Meilun Biotech Co., Ltd, Dalian, China). The lysate was centrifuged for 10 min at 12000 rpm/min at 4°C and the supernatant was obtained for further analysis. The total protein was measured by using BCA kit (Shanghai Yuan-mu Biotech Co., Ltd, Shanghai, China). Equal amounts of total protein were loaded onto a 10% SDS-PAGE gel and then transferred onto polyvinylidene fluoride membranes (PVDF, Dalian Meilun Biotech Co., Ltd, Liaoning, China) by using a wet transmembrane device. After blocking with 5% non-fat milk at room temperature for 1 h, the membranes were incubated overnight with primary antibodies followed by incubation with the appropriate HPR-conjugated secondary antibody for 2 h at room temperature. Then, the PVDF membranes were incubated with ECL reagent (Santa Cruz Biotechnology) in order to develop the blots. All values were normalized to β-actin. The primary antibodies PTEN (ab32199; 1:1000 dilution), Active-caspase 3 (ab2302; 1:1000 dilution) and β-actin (ab8227; 1:1000 dilution) were all obtained from Abcam (Cambridge, MA, USA). The second antibody was purchased from Cell Signaling Technology (Danvers, MA, USA; 7074; 1:2000).

### Animals study

3-4 weeks old male BALB/c nude mice were purchased from SLAC (Shanghai, China) and feed with normal food and water in pathogen-free conditions. All procedures were in accordance with the National Institutes of Health guide for the care and use of laboratory animals and were approved by situational Ethics Committee at The Affiliated Hospital of Guizhou Medical University. 16 nude mice were randomly divided into four groups (four per group), namely LN-18 pcDNA3.1-NC group, LN-18 pcDNA3.1-LINC00657 group, U-188 MG pcDNA3.1-NC group and U-188 MG pcDNA3.1-LINC00657 group. LN-18 and U-118 MG cells were transfected with pcDNA3.1-NC or pcDNA3.1-LINC00657, respectively. Physiological saline was used to suspend the transfected cells (LN-18 or U-118 MG) to a concentration of 5 × 10^7^ per microliter. And then, a volume of 100 μl cell suspension was injected subcutaneously into the left armpit of nude mice. The tumor volume was measured weekly using following formulate: length × width^2^ × 0.5. The tumors were weighed and recorded after incubation for 4 weeks.

Cellular apoptosis in tumor tissue was assessed using a terminal deoxynucleotidyl transferase dUTP nick end-labeling assay kit (TUNEL, Sigma-Aldrich). In brief, following dewaxing and hydration, tumor sections were digested with proteinase K for 30 min and labeled with a TUNEL reaction mixture for 2 h at 37°C. TUNEL apoptosis index was measured by the following method. Five positive views of each tumor section were calculated (× 200). More than 500 cells were calculated in one view of each tumor section. TUNEL apoptosis rate = positive staining cells in each view/total cells calculated in each view.

### Statistical analysis

Data was presented as mean ± SD. Three independent experiments were performed. SPSS 19.0 software (SPSS, Chicago, IL, USA) was used to do the statistical analysis. ANOVA analysis or unpaired t-tests were chosen for statistical comparison as appropriate. Significant index P < 0.05 was used.

## References

[1] Verhaak RG. Moving the needle: optimizing classification for glioma. Sci Transl Med. 2016; 8:350fs14. 10.1126/scitranslmed.aah4740 27488893

[2] Loch-Neckel G, Bicca MA, Leal PC, Mascarello A, Siqueira JM, Calixto JB. In vitro and in vivo anti-glioma activity of a chalcone-quinoxaline hybrid. Eur J Med Chem. 2015; 90:93–100. 10.1016/j.ejmech.2014.11.014 25461314

[3] Sun YC, Wang J, Guo CC, Sai K, Wang J, Chen FR, Yang QY, Chen YS, Wang J, To TS, Zhang ZP, Mu YG, Chen ZP. MiR-181b sensitizes glioma cells to teniposide by targeting MDM2. BMC Cancer. 2014; 14:611. 10.1186/1471-2407-14-611 25151861 PMC4155117

[4] Liu H, Li J, Koirala P, Ding X, Chen B, Wang Y, Wang Z, Wang C, Zhang X, Mo YY. Long non-coding RNAs as prognostic markers in human breast cancer. Oncotarget. 2016; 7:20584–96. 10.18632/oncotarget.7828 26942882 PMC4991477

[5] Bao MH, Li GY, Huang XS, Tang L, Dong LP, Li JM. Long non-coding RNA LINC00657 acting as miR-590-3p sponge to facilitate low concentration oxidized low-density lipoprotein-induced angiogenesis. Mol Pharmacol. 2018; 93:368–75. 10.1124/mol.117.110650 29436491

[6] Hu B, Cai H, Zheng R, Yang S, Zhou Z, Tu J. Long non-coding RNA 657 suppresses hepatocellular carcinoma cell growth by acting as a molecular sponge of miR-106a-5p to regulate PTEN expression. Int J Biochem Cell Biol. 2017; 92:34–42. 10.1016/j.biocel.2017.09.008 28919047

[7] Quinn JJ, Chang HY. Unique features of long non-coding RNA biogenesis and function. Nat Rev Genet. 2016; 17:47–62. 10.1038/nrg.2015.10 26666209

[8] Atif F, Yousuf S, Stein DG. Anti-tumor effects of progesterone in human glioblastoma multiforme: role of PI3K/Akt/mTOR signaling. J Steroid Biochem Mol Biol. 2015; 146:62–73. 10.1016/j.jsbmb.2014.04.007 24787660

[9] Babu R, Kranz PG, Agarwal V, McLendon RE, Thomas S, Friedman AH, Bigner DD, Adamson C. Malignant brainstem gliomas in adults: clinicopathological characteristics and prognostic factors. J Neurooncol. 2014; 119:177–85. 10.1007/s11060-014-1471-9 24838419 PMC4510613

[10] Mueller S, Phillips J, Onar-Thomas A, Romero E, Zheng S, Wiencke JK, McBride SM, Cowdrey C, Prados MD, Weiss WA, Berger MS, Gupta N, Haas-Kogan DA. PTEN promoter methylation and activation of the PI3K/Akt/mTOR pathway in pediatric gliomas and influence on clinical outcome. Neuro Oncol. 2012; 14:1146–52. 10.1093/neuonc/nos140 22753230 PMC3424210

[11] Parsa AT, Waldron JS, Panner A, Crane CA, Parney IF, Barry JJ, Cachola KE, Murray JC, Tihan T, Jensen MC, Mischel PS, Stokoe D, Pieper RO. Loss of tumor suppressor PTEN function increases B7-H1 expression and immunoresistance in glioma. Nat Med. 2007; 13:84–88. 10.1038/nm1517 17159987

[12] Guo W, Wang Q, Zhan Y, Chen X, Yu Q, Zhang J, Wang Y, Xu XJ, Zhu L. Transcriptome sequencing uncovers a three-long noncoding RNA signature in predicting breast cancer survival. Sci Rep. 2016; 6:27931. 10.1038/srep27931 27338266 PMC4919625

[13] Jimenez Jimenez AM, Ruttkay-Nedecky B, Dostalova S, Krejcova L, Michalek P, Richtera L, Adam V. Specific magnetic isolation of E6 HPV16 modified magnetizable particles coupled with PCR and electrochemical detection. Int J Mol Sci. 2016; 17:E585. 10.3390/ijms17050585 27164078 PMC4881435

[14] Cesana M, Cacchiarelli D, Legnini I, Santini T, Sthandier O, Chinappi M, Tramontano A, Bozzoni I. A long noncoding RNA controls muscle differentiation by functioning as a competing endogenous RNA. Cell. 2011; 147:358–69. 10.1016/j.cell.2011.09.028 22000014 PMC3234495

[15] Tay Y, Rinn J, Pandolfi PP. The multilayered complexity of ceRNA crosstalk and competition. Nature. 2014; 505:344–52. 10.1038/nature12986 24429633 PMC4113481

[16] Kallen AN, Zhou XB, Xu J, Qiao C, Ma J, Yan L, Lu L, Liu C, Yi JS, Zhang H, Min W, Bennett AM, Gregory RI, et al. The imprinted H19 lncRNA antagonizes let-7 microRNAs. Mol Cell. 2013; 52:101–12. 10.1016/j.molcel.2013.08.027 24055342 PMC3843377

[17] Liu XH, Sun M, Nie FQ, Ge YB, Zhang EB, Yin DD, Kong R, Xia R, Lu KH, Li JH, De W, Wang KM, Wang ZX. Lnc RNA HOTAIR functions as a competing endogenous RNA to regulate HER2 expression by sponging miR-331-3p in gastric cancer. Mol Cancer. 2014; 13:92. 10.1186/1476-4598-13-92 24775712 PMC4021402

[18] Wang J, Liu X, Wu H, Ni P, Gu Z, Qiao Y, Chen N, Sun F, Fan Q. CREB up-regulates long non-coding RNA, HULC expression through interaction with microRNA-372 in liver cancer. Nucleic Acids Res. 2010; 38:5366–83. 10.1093/nar/gkq285 20423907 PMC2938198

[19] An Y, Furber KL, Ji S. Pseudogenes regulate parental gene expression via ceRNA network. J Cell Mol Med. 2017; 21:185–92. 10.1111/jcmm.12952 27561207 PMC5192809

[20] Moraes LN, Fernandez GJ, Vechetti-Júnior IJ, Freire PP, Souza RW, Villacis RA, Rogatto SR, Reis PP, Dal-Pai-Silva M, Carvalho RF. Integration of miRNA and mRNA expression profiles reveals microRNA-regulated networks during muscle wasting in cardiac cachexia. Sci Rep. 2017; 7:6998. 10.1038/s41598-017-07236-2 28765595 PMC5539204

[21] Oliveto S, Mancino M, Manfrini N, Biffo S. Role of microRNAs in translation regulation and cancer. World J Biol Chem. 2017; 8:45–56. 10.4331/wjbc.v8.i1.45 28289518 PMC5329714

